# Prevalence of blindness and visual impairment among yanomami Indigenous people in the Brazilian Amazon region: a cross-sectional observational study at CASAI-Y

**DOI:** 10.1016/j.lana.2025.101161

**Published:** 2025-07-03

**Authors:** Maria Christina Chagas Ferreira, Marcos Antonio Pellegrini, Bianca Jorge Sequeira

**Affiliations:** Universidade Federal de Roraima, PROCISA, Boa Vista, RR, Brazil

**Keywords:** Yanomami, Indigenous people, Ocular health, Ophthalmology, Visual acuity, Blindness, Vision impairment, Public health

## Abstract

**Background:**

Indigenous populations in the Americas experience significant health inequities, especially in remote areas. Among the Yanomami, a highly mobile Indigenous people living in the Amazon, ocular health data are scarce. This study aimed to estimate the prevalence and causes of visual impairment and blindness among the Yanomami, contributing to public health planning for isolated populations.

**Methods:**

A cross-sectional observational study was conducted between June and August 2024 at the Yanomami Indigenous Health House (CASAI-Y), in Boa Vista, Brazil. A total of 158 self-identified Yanomami individuals aged ≥5 years were evaluated through comprehensive eye exams including presenting visual acuity (PVA), best-corrected visual acuity (BCVA), autorefractometry, dynamic refractometry, tonometry, fundus photography, and slit-lamp examination. Visual impairment was classified according to the ICD-11 criteria.

**Findings:**

The prevalence of moderate or worse visual impairment and blindness based on PVA was 15.8%, decreasing to 8.2% with BCVA. Cataract and uncorrected refractive error were the leading causes of visual loss. Visual impairment was more frequent among men and individuals aged >40 years. Statistically significant improvements in visual acuity were achieved with refractive correction (p < 0.001). Cultural and logistical barriers were identified as major limitations to access.

**Interpretation:**

The high burden of preventable visual impairment in the Yanomami population underscores the need for accessible and culturally adapted eye care services. Corrective lenses and cataract surgery could substantially improve quality of life. Targeted strategies including mobile screening, Indigenous health agents and teleophthalmology should be prioritized to prevent avoidable blindness.

**Funding:**

No funding.

**Research in context**

**Evidence before this study**

We searched PubMed, Scielo, and LILACS from database inception to January 2025 for studies reporting on ocular health, vision impairment, and blindness among Indigenous populations in Brazil, using the keywords “Yanomami” “Indigenous” “blindness” “visual impairment” “refractive error,” “cataract.” Although previous research has addressed specific infectious ocular diseases such as onchocerciasis and trachoma among the Yanomami, no prior study has systematically assessed the broader patterns of vision impairment and blindness or provided comprehensive ophthalmologic data for this population.

**Added value of this study**

This study provides the first systematic assessment of the prevalence of visual impairment and blindness among Yanomami Indigenous people. Our findings highlight a substantial burden of uncorrected refractive errors, cataract-related visual impairment, and pterygium, particularly among adults. The study also used a culturally adapted visual acuity charts specifically designed for the Yanomami population, enabling a cultural aligned visual assessment in remote Indigenous communities.

**Implications of all the available evidence**

The results of this study reveal important disparities in eye health among the Yanomami and emphasize the need for access to ophthalmologic care, including refractive services and surgical interventions for treatable causes of visual impairment. These findings underscore the importance of integrating routine eye health assessments into primary health care services for Indigenous populations living in remote areas. Furthermore, they contribute baseline data essential for planning public health strategies aimed at preventing avoidable blindness in vulnerable Indigenous communities.

## Introduction

Vision is the most dominant of the human senses. According to the World Health Organization (WHO), it plays a crucial role in all stages of life. However, numerous eye diseases affect the population, and many go undiagnosed or untreated, leading to avoidable visual impairment and blindness.^1^ Indigenous peoples are among the most underserved and marginalized populations worldwide. While they are diverse in language, culture, risk factors, and autonomy, they share common characteristics such as elevated poverty rates and increased burden of disease when compared to surrounding populations.^2^

In Brazil, the Indigenous population comprises approximately 1,694,836 individuals, accounting for 0.83% of the national population.^3^ Epidemiological data regarding Indigenous health are scarce and highly variable among different groups.^4^ Among minority Indigenous ethnic groups, the prevalence of monocular blindness can reach 10%, with cataract being common among older adults and refractive errors prevalent in children.^5^

The Yanomami are a culturally distinct Indigenous population characterized by high territorial mobility, traditionally moving across the Brazil–Venezuela border. They inhabit a continuous mountainous territory, encompassing parts of the states of Roraima and Amazonas in northern Brazil, with a history of recent and limited contact with non-Indigenous society.^6^ Currently, the Yanomami population in Brazil is estimated at 31,007 individuals, including approximately 5,893 children under five years old. They occupy the 9,664,975-hectare Yanomami Indigenous Territory, officially demarcated in 1992.^7^ The Yanomami Indigenous Territory is a restricted-access area, with entry permitted only to professionals from the Special Secretariat for Indigenous Health (SESAI) and partner institutions, in accordance with Joint Ordinance FUNAI/SESAI No. 1, dated January 30, 2023. The territory is a restricted-access area, with entry limited to authorized personnel, and access to most Yanomami communities is possible only by air (98%) or, to a very limited extent, by land (2%).^8^ Widely dispersed in remote regions, the Yanomami maintain minimal and selective contact with broader society, and despite the gradual expansion of permanent interactions in the current millennium, much of the population remains highly isolated due to the vast distances involved.^6^^,^^7^^,^^9^

In Brazil, the Yanomami Special Indigenous Health District (DSEI-Y), responsible for healthcare delivery to this population, includes the Yanomami Indigenous Health House (CASAI-Y), 37 Base Centres, and 78 Indigenous Primary Health Units (UBSIs). Multidisciplinary teams provide care both at health units and during community outreach visits. Access to the region is only possible via aircraft, helicopters, or extensive walking, resulting in highly complex and challenging logistics.^7^ Geographic isolation and the high mobility of the Yanomami population impose substantial challenges to the organization and delivery of health services across the territory.^10^

An extensive search of scientific databases (CAPES, Medline, Pubmed, Lilacs, Scielo and Cochrane) was conducted in April 2023 to support the development of this study. The search strategy, that involved combining the term “yanomami” with keywords related to ocular health, such as “blindness”, “visual impairment” “ocular” and “eye health”, revealed only twelve publications related to ocular health in the Yanomami population, the most recent dating from 2018. These studies focus on specific ocular diseases without addressing the broader prevalence or causes of visual impairment and blindness. The lack of up-to-date, population-level data on vision health among the Yanomami represents a significant epidemiological gap, hindering the development of public policies tailored to this population’s ocular healthcare needs.

To advance the current understanding of the Yanomami population’s vision health, a comprehensive study was designed and implemented.

Ideally, a population-based survey conducted directly within the Yanomami Indigenous Territory would have provided the most comprehensive assessment of visual health needs. However, such an endeavour is both financially prohibitive and logistically impractical. Conducting fieldwork would require the repeated transportation of ophthalmological equipment and specialized personnel under extremely challenging environmental conditions, demanding substantial financial resources. These geographic, legal, and logistical barriers make it virtually impossible to carry out a territory-wide survey with broad and equitable population coverage.

Given these constraints, the study was conducted at the Yanomami Indigenous Health House (CASAI-Y) in Boa Vista, capital of the State of Roraima, the most viable and logical alternative to ensure access to a diverse and representative sample of the Yanomami population. CASAI-Y is not a clinical facility but a support and accommodation centre for Yanomami individuals who must temporarily relocate to Boa Vista for health treatments or other essential needs. Reflecting their cultural practices, Yanomami patients usually travel accompanied by their immediate and extended family members, which results in a diverse and representative cross-section of the population at the facility. The temporary establishment of ophthalmological services within CASAI-Y, using equipment and personnel provided by the principal investigator, enabled the comprehensive evaluation of individuals temporarily residing there while awaiting transportation back to their communities.

This article presents findings derived from a previously defended and approved Master’s degree dissertation and focuses specifically on the prevalence of blindness and visual impairment among the Yanomami. The study aimed to evaluate the prevalence of blindness and visual impairment and to identify associated factors among a convenience sample of Yanomami individuals temporarily residing at CASAI-Y. The research is justified by the profound impact of vision on individual well-being and quality of life, particularly in communities with limited external contact and fragile economies, such as the Yanomami, where individuals with disabilities may face social stigma if unable to contribute to the group or defend themselves against environmental hazards.

## Methods

This was an observational, cross-sectional, descriptive, and analytical study with a quantitative approach. Upon receiving ethical approval from the Research Ethics Committee of the Federal University of Roraima and the Brazilian National Research Ethics Commission (Approval No. 6.479.368–CEP/CONEP), epidemiological and clinical data collection was initiated and conducted in full accordance with the principles of the Declaration of Helsinki.

Visual acuity (VA) was assessed based on both presenting visual acuity (PVA) and best-corrected visual acuity (BCVA), following the definitions established by the WHO. A comprehensive ophthalmological examination was also performed to identify the causes of blindness and visual impairment, as well as associated risk factors.

The study included self-identified individuals of the Yanomami ethnic group, aged 5 years or older, who were present at the Yanomami Indigenous Health House (CASAI-Y) during the data collection period. Participation was voluntary and based on informed consent; for minors, authorization was obtained from a legal guardian. Informed consent and/or assent were obtained in writing, with all documents translated into the participants’ native language and orally communicated by a native-speaking interpreter. Considering the high prevalence of illiteracy among participants, the consent process was also audio recorded to ensure full ethical compliance and participant comprehension. In cases where written consent could not be provided by the participant, the interpreter signed the consent form on their behalf, as per protocol.

Exclusion criteria included failure to meet inclusion criteria, presence of cognitive impairment, absence of minimal clinical conditions required for the examination, or refusal to participate in the study.

### Study population and sample

The target population consisted of individuals temporarily residing at the Yanomami Indigenous Health House (CASAI-Y), who had travelled from various regions within the Yanomami Special Indigenous Health District (DSEI-Y) to receive medical care, as well as their family members aged five years or older, all self-identified as Yanomami.

**Fig. 1:**
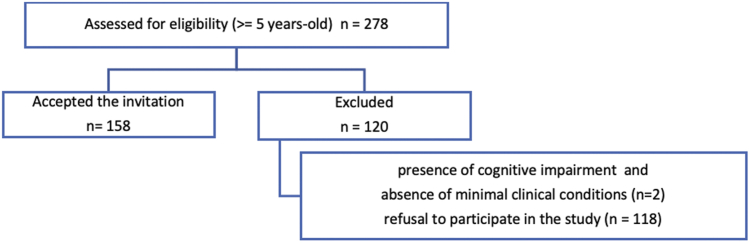
Flow diagram showing the recruitment and inclusion of participants in the study—2024.

During the data collection period, from June to August 2024, a total of 278 eligible individuals, older than 5 years-old, belonging to four Yanomami subgroups—Yanomami, Sanuma, Xirixana, and Xiriana—were present at the facility, representing the entire Yanomami population available for evaluation. The study adhered strictly to ethical principles, and participation was voluntary following invitation and explanation by interpreters. Following formal consent or assent, data were collected from 158 individuals who met the inclusion and exclusion criteria and agreed to participate in the study, accounting for 56.83% of the present population. The participant flow is detailed in the CONSORT diagram (Fig. 1). This convenience sample size meets the statistical requirement for a 95% confidence level and a 5% margin of error in a homogeneous population.

### Data collection

**Fig. 2:**
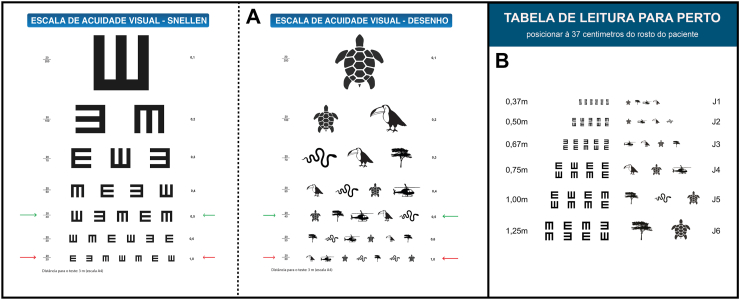
Visual acuity testing charts adapted for the study population. A—Distance charts: Snellen “E” and culturally adapted pictorial chart. B—Near vision charts: Jaeger “E” reading chart and culturally adapted pictorial version—2024.

Data collection was conducted over four scheduled visits and followed a three-stage ophthalmologic examination protocol. In stage one, basic demographic data—such as name, sex, age, home community, and base health post—were recorded, and informed consent or assent was obtained. Stage two involved objective data collection by trained technicians using a Welch Allyn Spot Vision Screener, a handheld photoscreener for autorefraction, a Huvitz HNT-7000 non-contact tonometer for intraocular pressure, and an Eyer portable fundus camera for retinal imaging. In stage three, immediately following the objective assessments, a clinical interview was conducted to gather patient-reported information on ocular history and general health, followed by a slit-lamp biomicroscopy examination using the Advanced portable slit lamp and subjective refraction testing by an ophthalmologist. Distance visual acuity was assessed with both a Snellen “E” chart (Brazil, Ministry of Health, 2008) and a culturally adapted pictorial chart developed specifically for the Yanomami, positioned at a 3-m distance (measured from the back legs of the chair) in a well-lit room. All examinations were conducted by the ophthalmologist. Each eye was examined separately without correction. For individuals with a presenting visual acuity (PVA) below 20/30 and self-reported visual complaints, the best-corrected visual acuity (BCVA) was assessed dynamically using a Greens refractor. Near visual acuity was also tested in individuals aged over 35 using a Jaeger near vision card featuring the “E” optotype and a corresponding pictorial version adapted for this study. The charts were positioned 37 cm from the patient’s face, and testing was again conducted with the Greens refractor. Interpreters were present throughout the interviews, exams, and refraction assessments, and were selected based on their fluency in the specific dialect spoken by the participant. There were no adverse events related to the use of cycloplegic agents. Participants were informed—through interpreters—about each procedure, including eye drops and ocular contact, and exams were only conducted upon verbal agreement. At the end of the examination, prescriptions for corrective lenses were provided when indicated. In cases requiring surgical treatment, patients were referred to the Brazilian Unified Health System (SUS) regulatory service, and all findings were documented in the patient’s medical record. A culturally adapted visual acuity screening kit was developed as an outcome of this research. It includes visual acuity charts tailored for the Yanomami population based on Ministry of Health guidelines (Fig. 2), an application manual and an instructional video recorded both in Portuguese and in the Yanomami language. This kit is available in digital format and is intended for use in local schools and by Indigenous health agents to support early detection of visual acuity loss that may require specialist care.

### Visual acuity classification

Visual acuity (VA) was classified according to the International Classification of Diseases 11th Revision (2018), which defines visual impairment in two groups—distance and near VA—using the Snellen chart. For distance, VA was categorized as follows: normal when VA ≥ 20/30; mild visual impairment, 20/30 < VA ≤ 20/60; moderate visual impairment, 20/60 < VA ≤ 20/200; and severe visual impairment, 20/200 < VA ≤ 20/400. VA < 20/400 was considered blindness. The primary cause of VA < 20/30 was reported for each eye. For near vision, impairment was defined as the inability to identify all 14 characters at the J1 level on the Jaeger chart positioned at 37 cm from the patient’s face; identifying at least one character at this level was considered normal near vision (Duarte et al., 2003; Khachatryan et al., 2024). Presenting visual acuity with best correction (PVA-BC) was also measured, but not used for impairment classification, as access to and acceptance of visual rehabilitation resources such as glasses or surgery could not be assured in this population; instead, it was used to assess the potential impact of interventions.^1^ Monocular vision was defined when one eye had normal VA while the fellow eye presented with SVI or blindness.

### Data analysis

Data were tabulated as collected during the ophthalmologic examination. Continuous variables were reported as means with standard deviations, while categorical variables were summarized as absolute frequencies and percentages. The prevalence of visual impairment and its underlying causes was presented in frequency distribution tables and graphical formats, with stratification by age group, sex, and subgroups. Risk factors for visual impairment were assessed through correlational analysis. Paired-sample Student’s t-tests were used for dependent variables (e.g., comparison between presenting visual acuity and best-corrected visual acuity), one-way ANOVA was applied for independent variables, and Pearson’s correlation coefficients were calculated for continuous variable associations. Additionally, Odds Ratios (OR) with 95% confidence intervals (CI) were computed for statistically significant associations. Data tabulation and statistical analysis were performed using Microsoft Excel, Statistica 12 software (TIBCO Software Inc.) and StatPlus (AnalystSoft) software.

#### Ethics approval

Research Ethics Committee of the Federal University of Roraima and the Brazilian National Research Ethics Commission (Approval No. 6.479.368–CEP/CONEP).

### Role of the funding source

The research received no specific grant from any funding agency.

## Results

### Participation response

According to 2023 demographic data, the Yanomami population comprises 31,007 individuals, with a balanced gender distribution of 48.96% (15,180) female and 51.04% (15,827) male.^7^ A similar distribution was observed among the 278 eligible individuals present at CASAI-Y during the study period, with 48.92% (136/278) female and 51.08% (142/278) male (Table 1). Although this distribution was balanced, a significant difference was observed in participation rates.

**Table 1:** Distribution of individuals examined at CASAI-Y by sex, age group, and subethnic subgroup (n = 158).

	Present			Examined		
	Female	Male	Total	Female	Male	Total
**Age (years)**						
0–19	38 (55.1%)	31 (44.9%)	69 (24.8%)	13 (40.6%)	19 (59%)	32 (20.2%)
20–39	66 (53.2%)	58 (46.8%)	124 (44.6%)	27 (40.3%)	40 (59.7%)	67 (42.4%)
40–59	27 (35.1%)	50 (64.9%)	77 (27.7%)	15 (26.3%)	42 (73.7%)	57 (36.1%)
+60	5 (62.5%)	3 (37.5%)	8 (2.9%)	1 (50.0%)	1 (50.0%)	2 (1.3%)
**Subgroups**						
Sanumã	44 (47.8%)	48 (52.2%)	92 (33.1%)	7 (18.4%)	31 (81.6%)	38 (24.0%)
Xiriana	5 (41.7%)	7 (58.3%)	12 (4.3%)	1 (20.0%)	4 (80.0%)	5 (3.2%)
Xirixana	17 (77.3%)	5 (22.7%)	22 (7.9%)	6 (54.6%)	5 (45.4%)	11 (7.0%)
Yanomami	70 (46.1%)	82 (53.9%)	152 (54.7%)	42 (40.4%)	62 (59.6%)	104 (65.8%)
**Total**	**136 (48.9%)**	**142 (51.1%)**	**278**	**56 (35.44%)**	**102 (64.56%)**	**158**

Among females, 41.2% (56/136) consented to participate in the study, compared to 71.8% (102/142) among males. Chi-square tests were performed to analyse participation response by sex. Same analysis was conducted across age groups and ethnic subgroups.

Participation patterns revealed that, overall, men were more likely to accept the invitation to undergo ophthalmological examination (Odds Ratio [OR] 3.64; 95% Confidence Interval [CI]: 2.20–6.00; p < 0.001). Statistically significant differences were observed in the 0–19 years (p = 0.045), 20–39 years (p = 0.003), and 40–59 years (p = 0.015) age groups, with males more likely to participate than females.

Trends varied among subgroups: individuals from the Yanomami subgroup exhibited higher acceptance rates compared to other subgroups, (OR 2.89; 95% CI: 1.69–4.96; p < 0.001), with no statistically significant difference between male and female participants. In contrast, the Sanumã subgroup were significantly less likely to participate (OR 0.39; 95% CI: 0.24–0.64; p < 0.001), with a statistically significant disparity between sexes (p < 0.001), as men were substantially more likely than women to consent to examination.

These findings may reflect cultural, social, or logistical factors influencing participation, such as differences in availability and decision-making autonomy. In Indigenous communities, women’s participation in healthcare initiatives is often shaped by caregiving responsibilities, limited control over health-related decisions, and structural barriers to accessing services.^11^

**Fig. 3:**
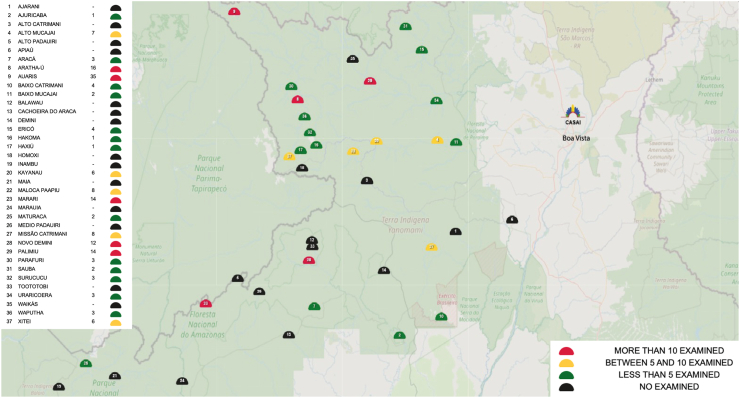
Distribution of patient consultations by base health post, stratified by number of individuals examined—2024.

The mean age of participants was 32.4 (13.8) years, with similar averages observed across sexes: 33.5 (13.74) for males and 30.4 (13.9) for females. The majority of individuals evaluated were within the 20–39-year age group. The mean age across subgroups followed a similar pattern, with values closely aligned with the overall sample mean: 32.7 (11.8) years for the Sanumã, 29.4 (12.5) years for the Xiriana, 32 (14.6) years for the Xirixana, and 32.5 (14.6) years for the Yanomami (Table 1). Participants in the study originated from 23 out of the 37 base health centres (Polos-Base) that comprise the Yanomami Special Indigenous Health District (DSEI-Y) (Fig. 3). These individuals represented 6 of the 7 macro-regions within the district, encompassing areas that account for approximately 91.11% of the total population of the Yanomami Territory.

An analysis was conducted comparing the distribution of consultations at CASAI-Y to the overall population distribution within the Yanomami Territory, using Pearson’s correlation coefficient, based on data from each base centre. A moderate positive correlation was observed between the number of individuals examined at CASAI-Y and the total population of their respective communities (Pearson’s correlation coefficient = 0.57, p = 0.0002). Adjusted population (age ≥5 years) analysis showed a strong and statistically significant positive correlation between the total Yanomami population and the number of eligible individuals present at CASAI-Y (r = 0.75, p < 0.001), suggesting that representation at the facility was proportional to the distribution of the broader Yanomami population across different regions. Additionally, a moderate and statistically significant correlation was also found between the total Yanomami population and the number of individuals who participated in the ophthalmological examination (r = 0.63, p = 0.001, n = 23).

Regarding age distribution, a Pearson correlation analysis revealed a moderate to strong positive association between the number of Yanomami individuals aged 10–59 years examined at CASAI-Y and the number of individuals from this age group who participated in the ophthalmological examination (r = 0.68, p < 0.001, n = 23). Regarding sex distribution, a chi-square test demonstrated a statistically significant association between the sex distribution of the study sample and that of the total population (p < 0.001).

Taken together, these positive correlations and associations, comparing the demographic characteristics of the Yanomami population in the territory and those of the sample attended at CASAI-Y, suggest that the sample presents a relevant degree of demographic alignment with the broader Yanomami population. This strengthens the potential to extrapolate the study findings to the general population living within the Yanomami Indigenous Territory.^12^

### Prevalence of blindness and visual impairment

Participants were evaluated based on their presenting visual acuity (PVA), assessed in the condition in which they presented. If wearing eyeglasses, vision was tested with their current correction. The vast majority of individuals (98.5%) were not wearing glasses at the time of the examination. All participants were then reassessed following dynamic refraction, and their results reclassified according to best-corrected visual acuity (BCVA). Based on PVA, 74.7% of participants were found to have normal vision, 9.5% had mild visual impairment, 9.5% had moderate visual impairment, 2.5% had severe visual impairment, and six individuals were legally blind. After dynamic refraction, the proportion of individuals with normal vision increased to 91.8%, with 1.9% remaining with mild visual impairment, 3.2% with moderate visual impairment, and no cases classified as severe visual impairment. A total of 3.2% of participants remained legally blind even after correction (Table 2).

**Table 2:** Distribution of patients classified according to presenting visual acuity (PVA) and best-corrected visual acuity (BCVA), by sex, age group, and subethnic subgroup (N = 158), 2024.

	Normal		Mild visual impairment		Moderate visual impairment		Severe visual impairment		Blindness	
	PVA	BCVA	PVA	BCVA	PVA	BCVA	PVA	BCVA	PVA	BCVA
**Sex**										
Female	48 (85.7%)	54 (96.4%)	3 (5.4%)	–	1 (1.8%)	–	2 (3.6%)	–	2 (3.6%)	2 (3.6%)
Male	70 (68.6%)	91 (89.2%)	12 (11.8%)	3 (3.0%)	14 (13.7%)	5 (4.9%)	2 (2.0%)	–	4 (3.9%)	3 (2.9%)
**Age group**										
0–19	31 (96.9%)	32 (100%)	1 (3.1%)	–	–	–	–	–	–	–
20–39	56 (83.6%)	63 (94.0%)	4 (6.0%)	1 (1.5%)	4 (6.0%)	–	–	–	3 (4.5%)	3 (4.5%)
40–59	31 (54.4%)	49 (86.0%)	10 (17.6%)	2 (3.5%)	10 (17.5%)	4 (7.0%)	3 (5.3%)	–	3 (5.3%)	2 (3.5%)
60–80	–	1 (50%)	–	–	1 (50.0%)	1 (50.0%)	1 (50.0%)	–	–	–
**Subgroup**										
Sanumã	24 (63.2%)	34 (89.5%)	3 (7.9%)	1 (2.6%)	8 (21.0%)	2 (5.3%)	1 (2.6%)	–	2 (5.3%)	1 (2.6%)
Xiriana	4 (80%)	5 (100%)	–	–	1 (20%)	–	–	–	–	–
Xirixana	10 (90.9%)	10 (90.9%)	–	–	–	–	–	–	1 (9.1%)	1 (9.1%)
Yanomami	80 (76.9%)	96 (92.3%)	12 (11.5%)	2 (1.9%)	6 (5.8%)	3 (2.9%)	3 (2.9%)	–	3 (2.9%)	3 (2.9%)
**Total**	**118 (74.7%)**	**145 (91.8%)**	**15 (9.5%)**	**3 (1.9%)**	**15 (9.5%)**	**5 (3.2%)**	**4 (2.5%)**	**-**	**6 (3.8%)**	**5 (3.2%)**

In the sex-stratified analysis of presenting visual acuity (PVA), men exhibited a lower proportion of normal vision (68.7%) compared to women (85.7%). When assessing best-corrected visual acuity (BCVA), women also showed better outcomes, with 96.4% achieving normal vision and only two cases of legal blindness, whereas among men, 89.2% achieved normal vision, but mild (1.9%) and moderate (2.9%) visual impairment persisted, along with three cases of blindness (2.9%). Statistical analysis confirmed that the prescription of refractive lenses was effective in improving visual acuity for both sexes, with significant improvements observed in men (p < 0.0001) and women (p = 0.00097). However, women presented a higher proportion of normal vision and lower rates of visual impairment overall. No statistically significant association was found between sex and blindness/severe visual impairment for either PVA or BCVA. Nonetheless, a significant association was identified between sex and normal PVA, indicating that Yanomami women had a significantly higher probability of presenting normal vision compared to men (OR 2.74; 95% CI: 1.16–6.47; p < 0.05).

In the age-group distribution, all children under 15 years old presented normal PVA. Up to age 19, 96.9% had normal PVA, and all reached 100% normal vision after correction (BCVA). Among participants aged 20 to 39, the majority (83.6%) presented with normal PVA, with some cases of mild and moderate impairment and three cases of blindness (4.5%). After correction, 94.0% in this age group achieved normal vision, one individual remained with mild visual impairment, and the blind participants retained their status. In the 40 to 59 age group, 54.4% had normal PVA, while 5.3% were legally blind. After best correction, 85.9% reached normal vision, 10.5% retained some level of visual impairment, and 3.5% remained blind. Among elderly participants, one presented with moderate visual impairment and another with severe visual impairment. Following refraction, one individual reached normal vision, while the other remained with moderate impairment.

Regarding near vision difficulties—classically associated with ageing—25.9% of participants were found to have presbyopia (Table 3), with the majority of cases falling within the 40–59-year age range. Although presbyopia appeared more frequently among male participants (30.4%) compared to female participants (17.9%), the difference was not statistically significant. Notably, early-onset presbyopia, occurring before age 40, was also identified in 5.9% of the study population. This finding reinforces the importance of providing corrective lenses to improve visual quality and facilitate full participation in community life.

**Table 3:** Distribution of patients by presence of presbyopia, stratified by age group (N = 158), 2024.

Age	Presbyopia		
	No	Yes	Total
0–19	32 (100%)	–	32
20–39	63 (94.03%)	4 (5.97%)	67
40–59	20 (35.09%)	37 (64.91%)	57
60–80	2 (100%)	–	2
**Total**	**117 (74.05%)**	**41 (25.95%)**	**158**

Analysis of visual acuity by age group revealed that the correction of refractive errors produced statistically significant improvements (p < 0.001) among individuals aged 20–39 and 40–59 years, suggesting that the response to eyeglass prescription may vary with age, with greater effectiveness observed in young and middle-aged adults. No association was found between age and blindness/severe visual impairment (SVI) for either presenting visual acuity (PVA) or best-corrected visual acuity (BCVA) (p = 0.90). However, a significant association was observed between age and normal PVA, indicating that Yanomami children and young adults had a significantly higher probability of achieving normal vision compared to older adults and elderly individuals (OR 3.16; 95% CI: 1.08–9.22; p < 0.05).

In the analysis by ethnic subgroup (Table 2), the Sanumã presented the lowest proportion of individuals with normal PVA (63.2%), followed by the Yanomami (76.9%). Cases of blindness, based on PVA, were observed among Sanumã (5.3%), Yanomami (2.9%), and Xirixana (9.1%) participants. Regarding BCVA, the Sanumã subgroup achieved 89.5% normal vision, followed by the Xirixana (90.9%), Yanomami (92.3%), and Xiriana (100%). No statistically significant association was found between subgroup and visual status (normal vision, visual impairment, or blindness) for either PVA or BCVA.

Refractive errors were responsible for 74.3% of the visual impairment cases identified. A paired t-test comparing PVA and BCVA demonstrated a statistically significant improvement after refraction (t = −6.20, p < 0.000001). Results showed that the mean PVA scores were significantly lower than BCVA scores, with statistically significant differences across all visual impairment categories, except blindness, thereby confirming the positive impact of refractive correction even in cases of moderate to severe visual impairment (Table 4).

**Table 4:** Mean and standard deviation of presenting visual acuity (PVA) and best-corrected visual acuity (BCVA), according to visual acuity classification (N = 158), 2024.

Classification	PVA–mean (sd)	BCVA–mean (sd)	Mean difference (95% CI)	p
Normal	0.90 (0.17)	0.17 (0.96)	0.73 (0.60–0.85)	<0.0001
Mild visual impairment	0.45 (0.08)	0.08 (0.80)	0.37 (0.29–0.45)	<0.0001
Moderate visual impairment	0.20 (0.06)	0.06 (0.73)	0.15 (0.10–0.20)	<0.0001
Severe visual impairment	0.05 (0.00)	0.00 (0.60)	0.05 (0.02–0.08)	<0.05
**Total**	**0.73 (0.33)**	**0.33 (0.88)**	**0.40 (0.32–0.49)**	**<0.0001**

### Main causes of blindness and visual impairment after BCVA assessment

After best refractive correction, 3.2% of participants were classified as bilaterally blind and an additional 3.2% presented monocular vision loss. The leading cause of visual impairment following refractive correction was cataract (38.9%), followed by fundoscopic abnormalities (22.22%). Among male participants, cataract was also the most frequent cause of visual impairment and blindness after correction (38.9%), followed by fundoscopic changes. No dominant cause was observed among female participants. In the younger age group, fundoscopic alterations were the most prominent cause (33.3%). Among individuals aged 40–59 years, cataract was the most prevalent cause of visual impairment (40%), followed by pterygium and fundoscopic abnormalities. Among the elderly, cataract accounted for 100% of all cases of visual impairment and blindness. The Sanumã and Yanomami subgroups identified cataract as the main contributing factor to visual impairment and blindness, whereas the Xirixana and Xiriana subgroups each reported a single case, attributed to corneal leucoma and fundoscopic alterations, respectively (Table 5).

**Table 5:** Distribution of causes of visual impairment after best-corrected visual acuity, by sex, age group, and subethnic subgroup (N = 18), 2024.

	Leucoma	Pterygium	Cataract	Uveitis	Retina	Other	Total
**Sex**							
Female			1 (33.3%)	1 (33.3%)		1 (33.3%)	3
Male	1 (6.7%)	3 (20.0%)	6 (40.0%)		4 (26.7%)	1 (6.7%)	15
**Age**							
0–19							
20–39		1 (16.7%)	1 (16.7%)		2 (33.3%)	2 (33.3%)	6
40–59	1 (10.0%)	2 (20.0%)	4 (40.0%)	1 (10.0%)	2 (20.0%)		10
60–80			2 (100%)				2
**Subgroup**							
Sanumã			3 (60.0%)		1 (20.0%)	1 (20.0%)	5
Xiriana					1 (100%)		1
Xirixana	1 (100%)						1
Yanomami		3 (27.3%)	4 (36.4%)	1 (9.1%)	2 (18.2%)	1 (9.1%)	11
**Total**	**1 (5.6%)**	**3 (16.7%)**	**7 (38.9%)**	**1 (5.6%)**	**4 (22.2%)**	**2 (11.1%)**	**18**

## Discussion

### Yanomami visual health in context

When evaluating visual impairment in a population, the World Health Organization (WHO) recommends using presenting visual acuity (PVA) as the primary indicator, as it reflects not only the physiological condition of the eyes but also the individual’s physical, social, and cultural environment, as well as their access to quality care, eyeglass prescription and provision, and rehabilitation opportunities.^1^ In the present study, only 1.43% of participants were using eyeglasses, and by evaluating both PVA and best-corrected visual acuity (BCVA), we were able to assess the real impact of limited access to visual correction through prescription lenses.

The results indicate that the ocular health status of Indigenous individuals evaluated at CASAI-Y is more concerning compared to other Indigenous populations previously studied in Brazil. Global estimates of the prevalence of visual impairment and blindness stood at 4.34% in 2020, based on WHO definitions encompassing moderate and severe visual impairment and blindness.^13^ In contrast, the present study identified a prevalence of 15.8% for the Yanomami population under the same criteria.

A study conducted in the Xingu region with riverside Indigenous communities^5^ from the Xingu National Park reported a 10% prevalence of mild, moderate, and severe visual impairment and blindness based on PVA—lower than the 25.3% found in the present study. That same study reported a 7.15% prevalence using BCVA, also below the rate found among the Yanomami (8.2%), as shown in Table 2. While the Xingu findings were considered, at the time, to reflect worse visual outcomes than other regions and Indigenous populations, the rates remain lower than those observed in the Yanomami population in this study.

Other studies of Indigenous populations worldwide have also explored the prevalence of visual impairment and blindness. In the present research, visual impairment and blindness affected 45.6% of participants over the age of 40—a rate considerably higher than those reported in other publications. A study among American Indian and Alaska Native populations reported a 12.8% prevalence of visual impairment among individuals over 40 years of age using PVA,^14^ while among Indigenous Australians the rate was 11.3%.^15^ The prevalence of blindness among participants over 40 years in the present study was 5.3%, higher than that reported in other Indigenous groups.^16^^,^^17^ Although direct comparisons should be interpreted with caution due to differences in study design and sampling methods,^18^ these findings suggest that the Yanomami participants in this study had poorer presenting vision than other Indigenous populations previously studied, including those living in similarly remote and hard-to-reach regions. The logistical and cultural challenges involved in adequately evaluating this population may explain the limited access to eye exams, eyeglasses, and surgical care, as healthcare teams face substantial difficulty providing regular services due to high population mobility and the region’s complex geography.^7^

Regarding sex-based differences, the prevalence of visual impairment among men was 19.61%, compared to 8.93% among women. Women also had a higher prevalence of normal PVA and BCVA, while men continued to experience visual impairment even after refractive correction, despite a positive effect from lens prescription in both sexes. The literature generally shows that men have a higher prevalence of vision loss due to trauma,^19^^,^^20^ while women more commonly present with visual impairment and blindness caused by age-related conditions such as cataract, and experience greater barriers to accessing healthcare.^13^^,^^21^ The divergent findings in our study are likely due to the younger profile of the sample, as complications requiring surgery or involving irreversible conditions tend to increase with age.

Additionally, female participants were markedly underrepresented in the study population. This imbalance may reflect sociocultural dynamics within Yanomami communities, where caregiving responsibilities, restrictions on mobility, and logistical barriers may limit women’s participation in health assessments outside urgent care contexts. Among the Yanomami, it is culturally common for women to exhibit shyness and to require authorization from their partners before engaging with non-Indigenous individuals, which may further restrict their participation in research activities.

Age distribution in the sample was also affected by cultural and logistical factors. The relocation of Yanomami individuals from their territories to CASAI-Y is coordinated by the federal government whenever specialized healthcare is needed, given the limited availability of healthcare services within Indigenous areas. However, elderly Yanomami often resist leaving their communities, as cultural beliefs emphasize the importance of dying within their territory and in accordance with traditional funeral rites.^22^ This cultural factor likely contributed to the small number of participants aged 60–80 years. Additionally, individuals with reduced mobility may face challenges in reaching base health centres from remote communities, limiting their ability to access referral pathways for specialized care.

Numerous studies have demonstrated that age is a key determinant in the risk of visual impairment among Indigenous populations.^5^^,^^23^, ^24^, ^25^ Our study showed that the vast majority of young Yanomami had good visual acuity, and that adults up to middle age benefited from simple interventions such as refractive correction. Further investigation is needed into the causes of blindness in young individuals, as 4.48% of participants under 40 and 5.51% of those over 40 had causes of blindness unrelated to uncorrected refractive error.

Studies in Indigenous populations have shown that visual impairment from uncorrected refractive error restricts individuals who would otherwise be healthy from performing daily community activities, such as crafts or navigating the forest, thus limiting their group participation. These studies emphasize the importance of providing corrective lenses to these populations.^23^^,^^25^ While Indigenous children may have superior visual acuity compared to non-Indigenous populations, economically active adults often exhibit poorer visual performance. This disparity, however, can be mitigated through simple actions such as vision screening, appropriate lens prescription, and free distribution of glasses.^5^

In the Yanomami region, geographic isolation poses a major challenge to diagnosing visual impairment and offering refractive correction. Screening performed by existing health teams and Indigenous teachers within the DSEI-Y could identify individuals needing specialized care, with tools such as telemedicine used to support individualized treatment plans. Various global initiatives have sought to improve eye health in remote areas. In India, a project is evaluating the use of rapid-assembly eyeglass frames^26^; in Australia, the University of Melbourne is exploring strategies to eliminate disparities in eye health between Indigenous and non-Indigenous populations by expanding access to specialist care^27^; in Canada, a customized optotype chart was developed for the Inuit population^28^; in our study, the optotype chart composed of culturally relevant pictograms used during data collection may represent an important first step toward preventing blindness in the Yanomami population.

This study also found that, even after optimal correction, some causes of visual impairment and blindness—such as cataract, pterygium, corneal leucoma, fundoscopic abnormalities, and uveitis—persisted. These results are in accordance with other populations. Among Indigenous Australians, cataract is the leading cause of blindness, and refractive error the main cause of visual impairment.^27^ Similar findings have been reported in Indigenous populations in Tibet, Malaysia, Taiwan, and Fiji.^16^^,^^23^^,^^29^^,^^30^

In our study, cataract accounted for 38.89% of visual impairment cases following refractive correction. Together with pterygium, these conditions were responsible for 55.52% of visual impairment among the Yanomami. Both require surgical treatment and appropriate postoperative care, underscoring the need to overcome barriers to access—especially those related to language and culture. In the Brazilian Amazon, a project involving specialist care and eye surgeries for riverside populations has been ongoing for several years in partnership with the Brazilian Navy. According to researchers involved, the leading cause of blindness in remote Amazonian areas is population isolation and neglect by public authorities.^31^ Studies on Indigenous populations have shown that factors such as lack of interpreters, negative past experiences, cultural beliefs, and fear of surgery hinder treatment acceptance.^25^^,^^32^ Therefore, eye health interventions in these regions should be carefully planned to ensure prompt treatment of diagnosed cases, prevent irreversible blindness, and secure safe surgical care and proper postoperative follow-up in the communities.

Among irreversible causes of blindness, fundoscopic scarring accounted for 22.22% of cases after best correction. A positive correlation was identified between trauma history and fundoscopic alterations, regardless of visual acuity status. Ocular trauma remains one of the leading causes of preventable blindness globally and poses even greater risk in remote populations due to delays in emergency care.^19^ Indigenous populations have been shown to have poorer outcomes following ocular trauma, with common causes being accidents and interpersonal violence.^20^ In a study among the Kadiweu people, ocular trauma was associated with vision loss and occurred exclusively among male individuals.^12^ Similarly, in our study, 85.71% of participants who reported ocular trauma were male, consistent with prior literature.

While a direct correlation between ocular trauma and visual impairment could not be conclusively established in this study, the presence of macular retinal scars — typically irreversible — combined with the significant delays in accessing specialized ophthalmological care among the Yanomami suggests that trauma may represent a relevant, yet underdiagnosed, cause of vision loss in this population.

Infectious causes must also be considered: although no active lesions compatible with trachoma or onchocerciasis were observed during the clinical examinations, the Yanomami population has a documented history of these diseases,^4^^,^^6^^,^^33^^,^^34^ as well as toxoplasmosis. One participant exhibited pterygium and conjunctival changes that were consistent with trachomatous scarring. In addition, some retinal cicatricial lesions were noted that could potentially be related to previous onchocerciasis; however, definitive confirmation of their aetiology was not possible given the available clinical data.

### Limitations

This study represents the first effort to systematically assess ocular health and visual impairment among the Yanomami people through a structured ophthalmological examination. While the achieved sample size was somewhat smaller than desired for optimal statistical precision for the overall Yanomami population–potentially limiting the statistical power the study–is adequate for the population at CASAI-Y, and this limitation does not substantially compromise the validity of the findings. CASAI-Y, the Indigenous Health Support House in Boa Vista, is the only facility serving the Yanomami population in the region, meaning that all individuals in transit for medical care are received at this site. Consequently, the population present at CASAI-Y during the study period constituted the full eligible population for research.

Participants voluntarily consented to the ophthalmological examination, a process that was unfamiliar to most, yet the demographic characteristics of those examined closely mirrored those of the broader Yanomami population. It is likely that the refusal of 120 individuals to participate was influenced by the historically compromised relationship between Indigenous and non-Indigenous populations, particularly following the public health emergency declared among the Yanomami in 2023.^35^ The invasion of Yanomami Territory by illegal miners not only caused severe environmental degradation, extinguishing traditional hunting grounds and leading to widespread malnutrition, but also contributed to the spread of numerous infectious diseases. This recent history of trauma, environmental destruction, and disease likely fostered mistrust toward non-Indigenous personnel, potentially affecting participation rates in healthrelated research initiatives.

Additionally, as the data reflect only individuals temporarily residing at a referral facility, they may not fully represent the ocular health status of Yanomami individuals living in more remote and less accessible villages. Cultural barriers may also have contributed to this limitation. These factors should be carefully considered when interpreting and generalising the findings.

Despite these constraints, positive correlations and statistically significant associations between the examined sample and the general Yanomami population support the representativeness of the data. This consideration is particularly important given the severe logistical barriers inherent to conducting population-based research across the Yanomami Territory, where access is highly restricted and conditions for fieldwork are exceptionally challenging.

When addressing the findings, we prioritized comparisons with studies that shared similar designs. However, the unique characteristics of the Yanomami population required a broader approach. The limited number of ophthalmological studies involving Indigenous populations in Brazil, the Americas, and globally made it necessary to include references with diferente methodologies to ensure a robust and meaningful discussion. The Yanomami differ markedly from other Indigenous groups in Brazil regarding the timing and nature of contact with broader society, geographic isolation, distance from urban centers, transportation barriers, and linguistic differences. Some subgroups even maintain “no permanent relations with national societies and minimal interaction with other Indigenous or non-Indigenous peoples^36^” reinforcing the need for careful contextualization when interpreting our findings.

### Final considerations

This study showed that visual impairment among the Yanomami is both significant and largely preventable. This population demonstrated substantially lower presenting visual acuity compared to other groups, including Indigenous populations, and stands to benefit considerably from corrective lens prescriptions, which could improve quality of life and functional capacity for daily tasks, preventing the social exclusion of individuals who would otherwise be able to fulfil important roles within their communities.

Men were more likely to participate in the health evaluation than women, possibly reflecting sociocultural restrictions that limit women’s access to services. Children under 15 years demonstrated normal presenting visual acuity, suggesting that systematic screening in this age group may not be an urgent priority for primary healthcare strategies. In contrast, young and middle-aged adults showed significant improvement with refractive correction, highlighting them as a target group for screening and early intervention programs.

Cataract and pterygium surgeries also represent impactful interventions, though their implementation must be carefully planned to respect cultural particularities and ensure not only successful clinical outcomes but also alignment with broader goals of blindness prevention and improved well-being. The study also identified patterns suggesting that the higher prevalence of visual impairment among men could be associated with occupational trauma, pointing to the necessity of incorporating trauma prevention initiatives into Indigenous healthcare planning.

Together, these findings are relevant for health policy planning and underscore the importance of integrating periodic refraction services, provision of corrective lenses, and access to cataract and pterygium surgery into the Indigenous healthcare system, always respecting cultural specificities and aiming to improve overall community well-being.

## Contributors

Maria Christina Chagas Ferreira–conceptualization, data curation, including access to raw data, formal analysis, investigation, methodology, project administration, resources, writing—original draft, and writing—review & editing and final responsibility for the decision to submit for publication.

Marcos Antonio Pelegrinni–conceptualisation, methodology, resources, supervision.

Bianca Jorge Sequeira–formal analysis, methodology, supervision, writing—original draft, and writing—review & editing.

## Data sharing statement

Due to the nature of this study involving Indigenous populations, individual participant data and associated documents cannot be shared without prior consultation and formal authorization from the communities involved. Any request for access to deidentified participant data and the data dictionary will require approval by the relevant Indigenous community leadership, submission and approval of a detailed research proposal, signing of a Data Access Agreement ensuring respect for Indigenous data governance, confidentiality, and responsible data use. At this time, there are no additional documents available for sharing.

Data sharing requests may be addressed to the corresponding author and each request will be reviewed individually, subject to community approval and ethical oversight.

## Editor note

The Lancet Group takes a neutral position with respect to territorial claims in published maps and institutional affiliations.

## Declaration of generative AI and AI-assisted technologies in the writing process

During the preparation of this work the author(s) used ChatGPT (OpenAI, San Francisco, CA) to support the editing of text and improvement of language clarity in English. After using this tool, the author carefully reviewed, verified, and edited all generated content as needed and takes full responsibility for the integrity and accuracy of the final version of the manuscript and the published content.

## Declaration of interests

All authors declare no conflicts of interest related to the content of this manuscript.

Specifically:

No financial support, provision of study materials, medical writing assistance, or article processing charges were received for the development of this manuscript.

The authors have no grants, contracts, royalties, consulting fees, honoraria, expert testimony payments, support for meetings or travel, patents, advisory board participation, leadership roles, stock ownership, or receipt of equipment, materials, or services to declare.

There are no other financial or non-financial interests relevant to the submitted work.

All authors have completed and submitted the ICMJE Disclosure Form and certify that they have answered every question without altering the wording of any questions on the form.

## Appendix A Supplementary data

Suplementary file
