# Supplementary material for: Prevalence of blindness and visual impairment among yanomami Indigenous people in the Brazilian Amazon region: a cross-sectional observational study at CASAI-Y
Source: Lancet Reg Health Am. 2025 Jul 3;48:101161. doi: 10.1016/j.lana.2025.101161 (PMC12270601; doi:10.1016/j.lana.2025.101161)
Supplement: Suplementary file [file mmc1.pdf]

PREVALENCE OF BLINDNESS AND VISUAL IMPAIRMENT AMONG YANOMAMI INDIGENOUS PEOPLE IN THE BRAZILIAN AMAZON REGION: A Clinic-Based Study at CASAI-Y

Yanomami Population by Base Center, Age Group, and Sex: Total Territory, CASAI-Y Presence, and Study Participants

YANOMAMI TERRITORY

POLO	FEMALE			MALE			TOTAL			FEM	MALE	TOTAL
	5 - 9 years	10 - 59 years	≥60 years	5 - 9 years	10 - 59 years	≥60 years	5 - 9 years	10 - 59 years	≥60 years	TOTAL	TOTAL	TOTAL
AJARANI	2	12	1	3	20	2	5	32	3	15	25	40
AJURICABA	45	128	10	56	141	13	101	269	23	183	210	393
ALTO CATRIMANI	30	82	6	24	87	7	54	169	13	118	118	236
ALTO MUCAJAI	54	215	11	58	218	14	112	433	25	280	290	570
ALTO PADAUIRI	17	67	3	19	84	3	36	151	6	87	106	193
APIAÚ	15	69	4	14	65	7	29	134	11	88	86	174
ARACÁ	26	74	8	12	87	5	38	161	13	108	104	212
ARATHA-Ú	55	260	19	71	212	25	126	472	44	334	308	642
AUARIS	385	1426	86	410	1492	83	795	2918	169	1897	1985	3882
BAIXO CATRIMANI	15	48	3	23	54	8	38	102	11	66	85	151
BAIXO MUCAJAI	29	95	4	37	107	2	66	202	6	128	146	274
BALAWAU	68	265	25	70	257	28	138	522	53	358	355	713
CACHOEIRA DO ARACA	9	46	5	9	41	7	18	87	12	60	57	117
DEMINI	20	69	8	28	64	12	48	133	20	97	104	201
ERICÓ	28	123	9	32	134	9	60	257	18	160	175	335
HAKOMA	65	196	15	58	230	19	123	426	34	276	307	583
HAXIÚ	93	320	16	95	288	25	188	608	41	429	408	837
HOMOXI	20	76	8	27	100	5	47	176	13	104	132	236
INAMBU	55	203	15	49	207	11	104	410	26	273	267	540
KAYANAU	22	120	6	24	105	5	46	225	11	148	134	282
MAIA	55	197	21	48	214	18	103	411	39	273	280	553
MALOCA PAAPIU	43	153	14	48	162	10	91	315	24	210	220	430
MARARI	65	209	16	67	250	19	132	459	35	290	336	626
MARAUIA	248	884	71	206	945	51	454	1829	122	1203	1202	2405
MATURACA	199	753	44	202	741	48	401	1494	92	996	991	1987
MEDIO PADAUIRI	80	262	17	83	337	16	163	599	33	359	436	795
MISSÃO CATRIMANI	67	312	21	104	309	37	171	621	58	400	450	850
NOVO DEMINI	86	325	37	98	378	31	184	703	68	448	507	955
PALIMIU	112	333	22	107	396	29	219	729	51	467	532	999
PARAFURI	47	152	10	43	155	3	90	307	13	209	201	410
SAUBA	29	119	12	28	89	8	57	208	20	160	125	285
SURUCUCU	262	843	59	331	788	33	593	1631	92	1164	1152	2316
TOOTOTOBI	39	148	9	41	152	10	80	300	19	196	203	399
URARICOERA	10	52	4	13	48	4	23	100	8	66	65	131
WAIKÁS	17	72	8	23	73	4	40	145	12	97	100	197
WAPUTHA	80	238	10	77	256	11	157	494	21	328	344	672
XITEI	174	626	42	249	650	46	423	1276	88	842	945	1787
TOTAIS	2666	9572	679	2887	9936	668	5553	19508	1347	12917	13491	26408

CASAI-Y

POLO	FEMALE			MALE			TOTAL			FEM	MALE	TOTAL
	5 - 9 years	10 - 59 years	≥60 years	5 - 9 years	10 - 59 years	≥60 years	5 - 9 years	10 - 59 years	≥60 years	TOTAL	TOTAL	TOTAL
AJARANI	0	1	0	0	0	0	0	1	0	1	0	1
AJURICABA	1	0	0	0	1	0	1	1	0	1	1	2
ALTO CATRIMANI	0	1	0	0	0	0	0	1	0	1	0	1
ALTO MUCAJAI	1	7	0	1	4	0	2	11	0	8	5	13
ALTO PADAUIRI										0	0	
APIAÚ										0	0	
ARACÁ	0	0	0	0	3	0	0	3		0	3	3
ARATHA-Ú	2	8	1	3	7	0	5	15	1	11	10	21
AUARIS	6	32	2	4	44	0	10	76	2	40	48	88
BAIXO CATRIMANI	0	3	0	1	1	0	1	4	0	3	2	5
BAIXO MUCAJAI	0	4	0	0	0	0	0	4	0	4	0	4
BALAWAU	0	2	0	0	1	0	0	3	0	2	1	3
CACHOEIRA DO ARACA												
DEMINI	0	1	0	0	0	0	0	1	0	1	0	1
ERICÓ	1	1	0	0	4	0	1	5	0	2	4	6
HAKOMA	0	0	0	0	1	0	0	1	0	0	1	1
HAXIÚ	0	0	0	0	1	0	0	1	0	0	1	1
HOMOXI	0	1	1	0	3	0	0	4	1	2	3	5
INAMBU												
KAYANAU	1	5	0	1	2	0	2	7	0	6	3	9
MAIA										0	0	
MALOCA PAAPIU	0	3	0	0	7	0	0	10	0	3	7	10
MARARI	0	3	0	0	11	0	0	14	0	3	11	14
MARAUIA												
MATURACA	0	2	0	0	0	0	0	2	0	2	0	2
MEDIO PADAUIRI												
MISSÃO CATRIMANI	0	5	0	1	3	2	1	8	2	5	6	11
NOVO DEMINI	0	5	0	0	9	0	0	14	0	5	9	14
PALIMIU	0	12	0	0	7	1	0	19	1	12	8	20
PARAFURI	0	3	0	0	0	0	0	3	0	3	0	3
SAUBA	0	2	0	0	3	0	0	5	0	2	3	5
SURUCUCU	1	5	0	2	4	0	3	9	0	6	6	12
TOOTOTOBI												
URARICOERA	1	4	1	0	3	0	1	7	1	6	3	9
WAIKÁS												
WAPUTHA	1	2	0	1	4	0	2	6	0	3	5	8
XITEI	0	4	0	0	2	0	0	6	0	4	2	6
TOTAIS	15	116	5	14	125	3	29	241	8	136	142	278

ATTENDED DURIG THE RESEARCH

POLO	FEMALE			MALE			TOTAL			FEM	MALE	TOTAL
	5 - 9 years	10 - 59 years	≥60 years	5 - 9 years	10 - 59 years	≥60 years	5 - 9 years	10 - 59 years	≥60 years	TOTAL	TOTAL	TOTAL
AJARANI												
AJURICABA	0	0	0	0	1	0	0	1	0	0	1	1
ALTO CATRIMANI												
ALTO MUCAJAI	0	2	0	1	4	0	1	6	0	2	5	7
ALTO PADAUIRI												
APIAÚ												
ARACÁ	0	0	0	0	3	0	0	3	0	0	3	3
ARATHA-Ú	2	5	1	3	5	0	5	10	1	8	8	16
AUARIS	1	4	0	0	30	0	1	34	0	5	30	35
BAIXO CATRIMANI	0	2	0	1	1	0	1	3	0	2	2	4
BAIXO MUCAJAI	0	2	0	0	0	0	0	2	0	2	0	2
BALAWAU												
CACHOEIRA DO ARACA												
DEMINI												
ERICÓ	0	1	0	0	3	0	0	4	0	1	3	4
HAKOMA	0	0	0	0	1	0	0	1	0	0	1	1
HAXIÚ	0	0	0	0	1	0	0	1	0	0	1	1
HOMOXI												
INAMBU												
KAYANAU	1	3	0	1	1	0	2	4	0	4	2	6
MAIA												
MALOCA PAAPIU	0	2	0	0	6	0	0	8	0	2	6	8
MARARI	0	3	0	0	11	0	0	14	0	3	11	14
MARAUIA												
MATURACA	0	2	0	0	0	0	0	2	0	2	0	2
MEDIO PADAUIRI												
MISSÃO CATRIMANI	0	3	0	1	3	1	1	6	1	3	5	8
NOVO DEMINI	0	3	0	0	9	0	0	12	0	3	9	12
PALIMIU	0	8	0	0	6	0	0	14	0	8	6	14
PARAFURI	0	3	0	0	0	0	0	3	0	3	0	3
SAUBA	0	0	0	0	2	0	0	2	0	0	2	2
SURUCUCU	0	1	0	0	2	0	0	3	0	1	2	3
TOOTOTOBI												
URARICOERA	0	2	0	0	1	0	0	3	0	2	1	3
WAIKÁS												
WAPUTHA	0	1	0	0	2	0	0	3	0	1	2	3
XITEI	0	4	0	0	2	0	0	6	0	4	2	6
TOTAIS	4	51	1	7	94	1	11	145	2	56	102	158
